# Medical residents and COVID-19

**DOI:** 10.1192/j.eurpsy.2022.995

**Published:** 2022-09-01

**Authors:** R. Jomli, H. Jemli, U. Ouali, A. Maktouf, Y. Zgueb, A. Aissa

**Affiliations:** 1 Razi Hospital, Psychiatry A, manouba, Tunisia; 2 University of tunis elmanar, Faculty Of Medicine Of Tunis, manouba, Tunisia

**Keywords:** Stress, resident, hospital, covid

## Abstract

**Introduction:**

The covid-19 pandemic is a difficult global phenomenon that causes a lot of anxiety and uncertainty. This situation has involved reactions of fear. Healthcare professionals are necessarily in contact with patients, but may find themselves torn between the duty to care and the duty to protect themselves and their relatives.

**Objectives:**

To assess perceived stress among medical residents in Tunisia

**Methods:**

We conducted a descriptive study among a representative sample of residents working at a teaching hospital in Tunis during the first half of 2021 in different departments. We prepared a questionnaire for the study divided in two parts: socio-demographic data; professional data (function, practice setting); data related to contact with covid-19 patients ; questions on fear of covid-19 contamination and the Perceived stress scale (10items)

**Results:**

Our sample consists of 100 residents in 10 different specialties, including 70 in services with direct contact with Covid-19 patients. Stress management is rated good for 30 residents, average for 40 residents and poor for 30 residents. This management depends on the number of guards, the number of patients examined, the technical platform available and especially the period of the pandemic.

**Conclusions:**

Medical residents are in the front line in university hospitals in tunisia. The stress to which they are subjected depends on the working conditions and coping skills of each of them.

**Disclosure:**

No significant relationships.

